# Prevalence, knowledge, attitudes, and practices regarding Chagas disease in Guanare, Venezuela: a cross-sectional study

**DOI:** 10.1186/s13071-025-06846-4

**Published:** 2025-06-08

**Authors:** Iván A. Escalante-Pérez, Ana A. Alviares, Óscar D. Omaña-Ávila, Fhabián S. Carrión-Nessi, Daniela L. Mendoza-Millán, Grecia de J. Erimee-Vieira, Juan M. Contreras-Rengifo, Vanessa C. Sande-Mujica, Mariana de J. de Marchis-Vento, Karim J. Gebran-Chedid, Mario A. Dubuc-Ponte, Daniela I. Castro-Betancourt, Vittoria F. Fuentes-Fiore, Rachell A. Molina-Mendoza, Alejandro M. Loreto-Rodrigues, Juan C. Gomes-González, Luciano Mauriello, Eyleen Moronta, Belkisyolé Alarcón de Noya, Zoraida Díaz-Bello, David A. Forero-Peña

**Affiliations:** 1 “Luis Razetti” School of Medicine, Universidad Central de Venezuela, Caracas, Venezuela; 2 Vive Más Foundation, “Luis Razetti” School of Medicine, Universidad Central de Venezuela, Caracas, Venezuela; 3 Biomedical Research and Therapeutic Vaccines Institute, Ciudad Bolívar, Venezuela; 4 Immunology Section, Instituto de Medicina Tropical “Dr. Félix Pifano”, Universidad Central de Venezuela, Caracas, Venezuela; 5 Department of Internal Medicine, New York Medical College/Metropolitan Hospital Center, New York, NY USA; 6 Department of Infectious Diseases, Hospital Universitario de Caracas, Caracas, Venezuela

**Keywords:** Prevalence, Knowledge, Attitude, Practice, Chagas disease, Venezuela, Cross-sectional study

## Abstract

**Background:**

Chagas disease (CD) is endemic in 21 Latin American countries, placing approximately 75 million people at risk of infection. In Venezuela, CD has been recognized since 1919, with seroprevalence estimates reaching up to 45% between 1958 and 1968. However, current data on the epidemiology of CD in Venezuela are limited.

**Methods:**

We conducted a cross-sectional study in September 2023 in Guanare municipality, located in northwestern Portuguesa state. Seroprevalence was determined by detecting anti-*Trypanosoma cruzi* IgG antibodies and assessing specific-IgG avidity enzyme-linked immunosorbent assay (ELISA). Additionally, we evaluated knowledge, attitudes, and practices (KAPs) regarding CD in the region.

**Results:**

A total of 388 participants were enrolled, with a mean age of 29 (standard deviation [SD] = 21) years; 67% (*n* = 260) were female. Seven individuals, aged between 62 and 75 years, tested positive for *T. cruzi*, yielding an estimated seroprevalence of 2%. Among these ELISA-confirmed cases, the majority were men (71%, *n* = 5) with a mean age of 67 (SD = 5) years. Their occupations included construction work (42%, *n* = 3), farming (29%, *n* = 2), and housekeeping (29%, *n* = 2). In the KAP survey, 28% of respondents reported having a family member with CD, and 39% recalled observing the insect vector in their homes. Notably, 83% were aware that CD is transmitted by an insect vector. Although over 70% expressed positive attitudes toward educational initiatives and indicated willingness to collaborate with health services for prompt diagnosis and vector elimination, more than 80% reported inadequate vector control practices.

**Conclusions:**

The seroprevalence of CD in the Guanare population was 2%, with all positive cases occurring in individuals over 60 years of age, suggesting an absence of active transmission in the area. While community knowledge and attitudes regarding CD were generally favorable, vector control practices were suboptimal. Future studies employing probabilistic sampling across various regions of the state are needed to further elucidate the epidemiology of CD in Venezuela.

**Graphical Abstract:**

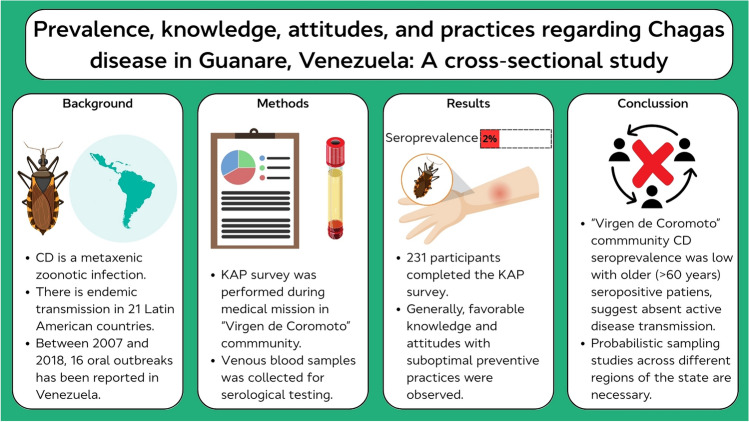

**Supplementary Information:**

The online version contains supplementary material available at 10.1186/s13071-025-06846-4.

## Background

Chagas disease (CD) is a metaxenic zoonotic infection caused by the hemoparasite *Trypanosoma cruzi*. In 2017, an estimated 6–7 million people were infected worldwide, with 30,000–40,000 new cases and 10,000 deaths annually [1, 2]. Historically, CD has been associated with low socioeconomic status, rural or jungle residences, and housing constructed with materials such as straw, wood, adobe, or bahareque. More recently, the disease has also been linked to the migration of populations from rural to urban areas and the adaptation of vectors to urban peripheries, a shift driven by deforestation and urbanization processes [3]. CD is endemic in 21 Latin American countries, placing approximately 70 million people at risk, particularly in Bolivia, Venezuela, Argentina, Brazil, and Mexico, where vector-borne transmission predominates [4–11]. Additionally, oral transmission remains a critical mode of infection; in some regions of Brazil (e.g., the Amazon basin), up to 70% of cases have been attributed to this route [12–14].

The acute phase of CD, which is often unrecognized, typically occurs 1–2 weeks after parasite exposure and is characterized by fever, myalgia, palpitations, rash, abdominal pain, and diarrhea, usually resolving within 4–8 weeks [15, 16]. Although myocarditis, meningoencephalitis, or death occur in fewer than 1% of patients during this phase, outbreaks involving oral transmission have reported morbidity and mortality rates exceeding 5% [17–19]. This acute phase is followed by an indeterminate phase, during which patients are asymptomatic. However, this phase may eventually progress to chronic manifestations, including chagasic cardiomyopathy and gastrointestinal complications such as megacolon and megaesophagus [15]. Serological assays are commonly employed to diagnose the indeterminate form of chronic CD.

In Venezuela, CD has been recognized since its initial description in 1919, with seroprevalence rates reaching up to 45% between 1958 and 1968 [20]. However, current epidemiological data are limited: the last national report was published nearly 8 years ago [21]. Regional studies conducted between 2003 and 2018 have estimated a prevalence of approximately 11% [22]. Moreover, oral transmission has led to 16 outbreaks between 2007 and 2018, affecting at least 321 individuals [23]. The primary mechanisms of oral transmission involve the consumption of food, beverages, or fruits contaminated with feces from infected triatomine bugs, particularly when such items are prepared in areas where human activities encroach upon natural parasite habitats [17].

To further elucidate the epidemiology of CD and assess community awareness in Venezuela, the present study was designed to determine the seroprevalence of CD and to evaluate the knowledge, attitudes, and practices (KAPs) related to the disease in an endemic community. The study was conducted in Virgen de Coromoto, a community in Portuguesa state that benefits from specialized healthcare provided during medical missions by the Vive Más Foundation—a nongovernmental organization affiliated with the Universidad Central de Venezuela.

## Methods

### Study area

**Fig. 1:**
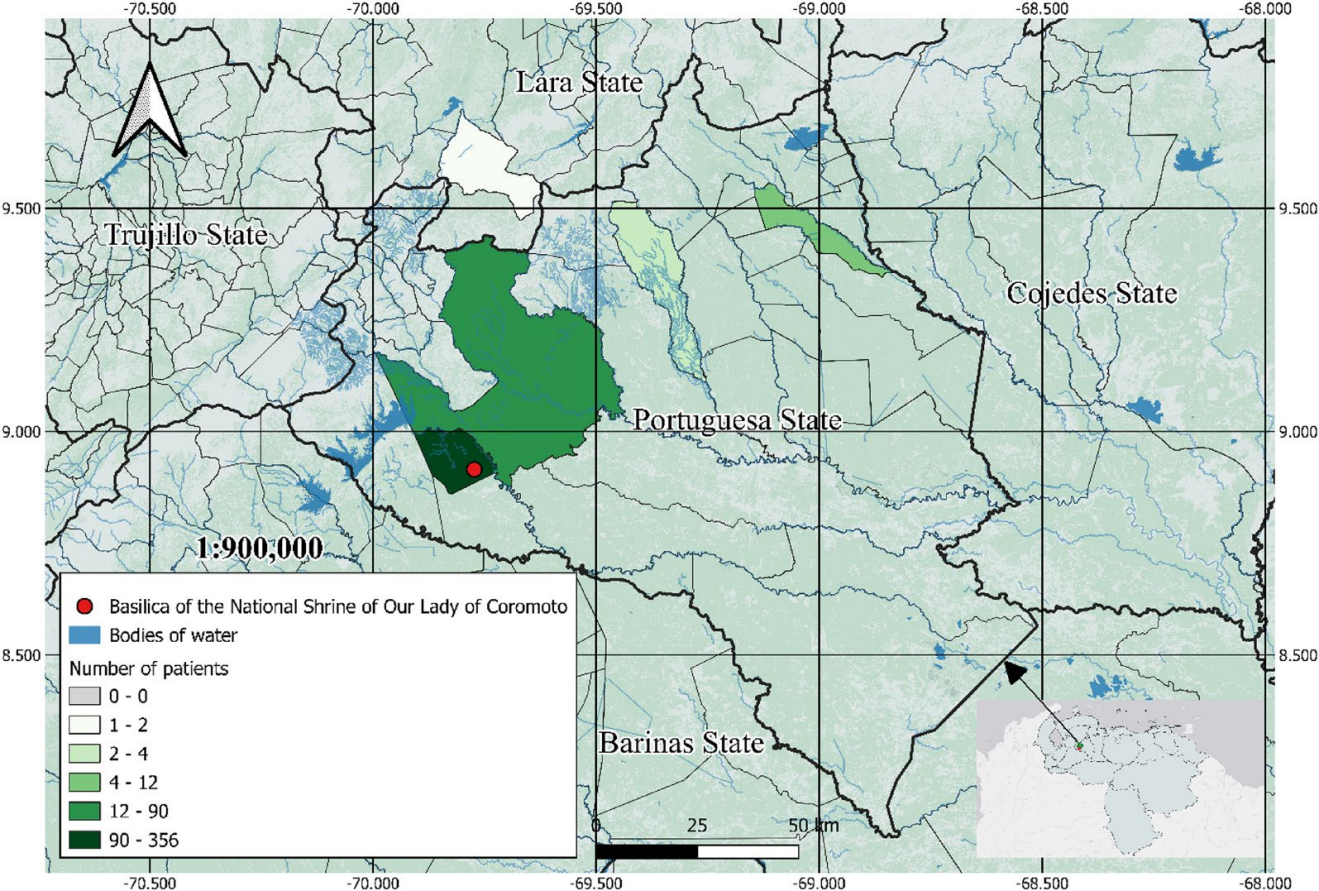
Patient origin map with the location of the medical mission headquarters (red), Basilica of the National Shrine of Our Lady of Coromoto, Guanare municipality, Portuguesa state, Venezuela

Venezuela, located on the northern coast of South America, covers approximately 916,445 km^2^. This study was conducted in Guanare municipality, situated in the northwestern part of Portuguesa state (central-western Venezuela), between the coordinates 08°52′36″ to 09°26′44″ N and 69°25′55″ to 69°58′50″ W. Guanare spans 2,008 km^2^, representing 13% of the state’s total area (Fig. 1). The study was conducted in Virgen de Coromoto community, which comprises 5,725 inhabitants and is located 11 km east of Guanare city. According to the 1990 census [24], 5,318 houses in the community are classified as farmhouses or rural dwellings (24% of the town’s homes); among these, 4,087 (19%) have outer walls constructed of adobe and wattle without additional framing, 2,977 (13%) have dirt floors, and 689 (3%) have roofs made of palms or reeds.

### Study design and population

A cross-sectional study was conducted in September 2023 in Virgen de Coromoto, with the Basilica of the National Shrine of Our Lady of Coromoto serving as the medical mission headquarters (Fig. 1). The research team, 1 month prior to data collection, in collaboration with medical specialists from the Vive Más Foundation, visited the community alongside local leaders and healthcare workers to invite all residents to participate. Both healthy and symptomatic individuals, regardless of clinical status, were invited to undergo diagnostic testing for CD. Prior to fieldwork, community leaders and healthcare workers received a detailed explanation of the study’s methodology, objectives, and scope, as well as the significance of community engagement. The study was promoted through biweekly meetings with household heads and during routine weekly home visits by local healthcare workers. Blood sampling for CD testing was performed after obtaining signed informed consent from adult participants; for those under 18 years old, consent was provided by a legal representative.

### Serology

For serological testing, 3 mL of venous blood was collected from each participant in sterile tubes without additives. The samples were collected in the field, allowed to sit, then centrifuged, and the serum was transferred to 2 mL conical plastic tubes containing 0.5% sodium azide. The serum was stored in freezers at –22 °C until transport in dry ice boxes to the laboratory where they were analyzed. The enzyme-linked immunosorbent assay (ELISA) for detecting anti-*T. cruzi* IgG antibodies was standardized and modified on the basis of the method of Voller et al. [25, 26] and subsequently modified by Díaz-Bello et al. [27]. Briefly, polystyrene plates (Nunc MaxiSorp™ ELISA, Thermo Fisher Scientific, Waltham, MA, USA) were coated with 50 μL of *T. cruzi* epimastigote antigen at a concentration of 10 μg/mL. Serum samples (50 μL) were added in duplicate at a 1:50 dilution and incubated at 37 °C for 30 min. Plates were washed three times with phosphate-buffered saline containing 0.05% Tween 20 (pH 7.2), after which 50 μL of human IgG conjugated to alkaline phosphatase (diluted 1:1000) was added and incubated for an additional 30 min at 37 °C. Following three further washes, 50 μL of substrate—p-nitrophenyl phosphate diluted in diethanolamine buffer (pH 10, 1 mg/mL)—was added and incubated at 37 °C for 15 min. The reaction was terminated with 50 μL of 1 N NaOH, and the optical density was measured at 405 nm using a Spectra Classic Plate Reader (Tecan, Männedorf, Switzerland). Optical density values after blank correction exceeding a cut-off of 0.2 were considered reactive.

Additionally, an avidity assay for CD was performed to estimate the time of infection in ELISA-positive patients by adapting methods previously utilized for toxoplasmosis [28, 29] (manuscript in preparation).

### KAP survey

A KAP survey was administered to community members aged 18 years or older, predominantly including household heads and primary income earners. The questionnaire was developed on the basis of established guidelines from previous studies [30–32] and was administered in Spanish. A panel of medical experts specializing in epidemiology, infectiology, parasitology, and research methodology validated the survey for practicality, feasibility, and relevance. A pilot test was conducted one month prior with 33 individuals (mean age = 42 years, standard deviation [SD] = 19; 79% female, *n* = 26) to refine the instrument.

The final questionnaire comprised 37 closed-ended questions organized into four sections: knowledge, attitudes, practices, and sociodemographic characteristics. The knowledge section included 10 multiple-choice questions (with options: yes, no, or I do not know) addressing CD transmission, symptoms, and treatment. The attitudes section consisted of five questions measured on a 5-point Likert scale (ranging from “strongly agree” to “strongly disagree”), and the practices section included two questions assessed on a 5-point frequency scale (ranging from “always” to “never”). The sociodemographic section contained 20 questions with both dichotomous (yes/no) and multiple-choice responses tailored to specific characteristics. Training workshops were conducted for the research team, and to ensure accurate identification of the disease vector, respondents were shown photographs of triatomine specimens (*Rhodnius prolixus*, *Panstrongylus geniculatus*, and *Triatoma maculata*).

### Statistical analysis

Descriptive statistics were used to summarize the data, including mean and SD, confidence intervals (CI), median and interquartile range (IQR), and frequencies with percentages (%). The normality of numerical variables was assessed using the Kolmogorov–Smirnov test. Statistical analyses were performed using SPSS version 27 (IBM Corp., Armonk, NY, USA), and figure was generated using.

## Results

### Sociodemographic characteristics and prevalence

A total of 388 participants were enrolled in the study (Table 1). The mean age was 29 (SD = 21) years, and 67% (*n* = 260) were female. Overall, seven participants (2%) tested positive for *T. cruzi* infection. Among participants residing in Virgen de Coromoto (88%, *n* = 357), six individuals (2%, 95% CI = 0.421–0.996) were positive. In contrast, among participants from Guanare (8%, *n* = 31), one individual (3%, 95% CI = 0.004–0.579) tested positive.

**Table 1:** Demographic characteristics of the study participants

Variable	All participants (*n* = 388, 100%)	ELISA IgG *T. cruzi* (*n* = 7)	95% CI
Age, mean (SD), years	29 (21)	67 (5)	–
Age grouped in years, *n* (%)			
< 10	95 (24.5)	0 (0)	–
10–20	62 (16)	0 (0)	–
21–40	94 (24.2)	0 (0)	–
41–60	102 (26.3)	0 (0)	–
> 60	35 (9)	7 (20)	0.59–1
Sex, *n* (%)			
Female	260 (67)	2 (0.8)	0.037–0.71
Male	128 (33)	5 (3.9)	0.29–0.963
Occupation, *n* (%)			
Student	127 (32.7)	0 (0)	–
Housekeeper	94 (24.2)	2 (21.2)	0.037—0.71
Construction worker	45 (11.6)	3 (6.7)	0.099—0.816
Farmer	28 (7.2)	2 (7.1)	0.037- 0.71
Teacher	22 (5.7)	0 (0)	–
Administrator	16 (4.1)	0 (0)	–
Businessman	13 (3.4)	0 (0)	–
Chef	5 (1.3)	0 (0)	–
Healthcare worker	4 (1)	0 (0)	–
N/A	34 (8.8)	0 (0)	–
Community, *n* (%)			
Virgen de Coromoto	357 (82)	6 (1.7)	0.421–0.996
Guanare	31 (8)	1 (3.2)	0.004–0.579

ELISA-confirmed positive cases were predominantly male (71%, *n* = 5) with a mean age of 67 (SD = 5) years. Their occupations included construction work (43%, *n* = 3), farming (29%, *n* = 2), and housekeeping (29%, *n* = 2). In nearly all positive cases (85%), the antibody avidity was greater than 60% (Supplementary Data 1 contains detailed avidity and optical density values).

### KAP survey

The KAP survey was completed by 231 participants, of whom 75% were female. Most respondents had completed high school (36%) or university (30%) education (Table 2). Among participants, 28% reported having a family member with CD, 39% had observed the vector in their home, and 7% reported being bitten by the vector; out of those bitten, only 20% sought medical attention.

**Table 2:** Demographic and epidemiological characteristics of the participants who answered the survey on KAPs

Characteristics	All (*n* = 231, 100%)
Age, mean (SD), years	43 (15)
Sex, *n* (%)	
Female	173 (74.9)
Male	58 (25.1)
Highest educational degree, *n* (%)	
None	14 (6.1)
Primary school	64 (27.7)
High school	84 (36.4)
University	69 (29.9)
Occupation, *n* (%)	
Housekeeper	87 (37.7)
Construction worker	34 (14.7)
Teacher	29 (12.6)
Farmer	26 (11.3)
Administrator	21 (9.1)
Student	18 (7.8)
Businessman	10 (4.3)
Chef	6 (2.6)
Have you seen the kissing bug (*chipo*) at home?, yes (%)	89 (38.5)
Have you ever been bitten by the bug?, yes (%)	15 (6.5)
After the bite, did you attend a healthcare facility?, yes (%)	3 (20)
Were you diagnosed with Chagas disease?, yes (%)	0 (0)
Household living surroundings, yes (%)	
Vegetation	203 (87.9)
Pets	178 (77.1)
Common opossum	141 (61)
Palms	119 (51.5)
Have you ever received a transfusion?, yes (%)	34 (14.7)
Have you ever donated blood?, yes (%)	41 (17.7)
Have you ever had a transplant?, yes (%)	1 (0.4)
Do you frequently drink natural juices at home?, yes (%)	125 (54.1)
Do you eat food derived from palms?, yes (%)	184 (79.7)
Do you have family members with diagnosis of Chagas disease?, yes (%)	65 (28.1)
Positive serology result (%)	7 (3)

Regarding their living environment, 88% of respondents noted abundant vegetation around their homes, 77% kept pets, and 61% reported observing *Didelphis marsupialis* (common opossum) in their backyard, nearby trees, or streets.

The knowledge section of the survey revealed that 83% of respondents were aware that CD is transmitted by an insect vector, 49% recognized that blood transfusion is a transmission route, and only 23% knew about vertical transmission. Additionally, 18% reported that CD could be transmitted through sexual intercourse, while 35% thought it might be spread via direct contact with dogs and cats. Regarding clinical manifestations, 78% knew that the infection could lead to cardiomegaly, and 55% recognized that the disease may be asymptomatic (Table 3).

**Table 3:** Knowledge survey results

Knowledge	All (*n* = 231, 100%)
How is Chagas disease transmitted?, correct (%)	
Through bugs (vectors)	191 (82.7)
From mother to child during delivery	53 (22.9)
Through blood transfusion	113 (48.9)
Through sexual relationships	41 (17.7)
Through physical contact with cats or dogs	81 (35.1)
Is it possible to acquire Chagas disease without presenting symptoms?, correct (%)	126 (54.5)
Can Chagas disease cause heart enlargement?, correct (%)	181 (78.4)
Is the vector for Chagas disease known as *chipo* in Venezuela?, correct (%)	204 (88.3)
The following represent characteristics for vector refugial at your home: palm roofs, mud walls, crackles in the walls?, correct (%)	180 (77.9)

Attitudinal responses indicated that over 70% of participants had positive perceptions regarding educational lectures and support for prompt diagnosis and vector elimination efforts through the healthcare system (Table 4). In terms of practices, more than 80% of respondents reported inadequate vector control measures (Table 5).

**Table 4:** Attitude survey results

Attitudes	All (*n* = 231, 100%)
Chagas disease is severe, *n* (%)	
Completely agree	129 (55.4)
Agree	58 (25.1)
Neutral	28 (12.1)
Disagree	16 (6.9)
Completely disagree	1 (0.4)
Would you like to be screened and tested for Chagas disease?, *n* (%)	
Completely agree	184 (79.7)
Agree	46 (19.9)
Neutral	0 (0)
Disagree	0 (0)
Completely disagree	1 (0.4)
If you find the disease vector (*chipo*) at home, would you take it to the nearest medical facility?, *n* (%)	
Completely agree	120 (51.9)
Agree	43 (18.6)
Neutral	23 (10)
Disagree	19 (8.2)
Completely disagree	26 (11.3)
Attending lectures on the disease could prevent you from acquiring Chagas disease?, *n* (%)	
Completely agree	149 (64.5)
Agree	59 (25.5)
Neutral	10 (4.3)
Disagree	10 (4.3)
Completely disagree	3 (1.3)
The best next step following a bite from the Chagas disease vector is to attend the nearest medical facility?, *n* (%)	
Completely agree	187 (81)
Agree	34 (14.7)
Neutral	4 (1.7)
Disagree	5 (2.2)
Completely disagree	1 (0.4)

**Table 5:** Practice survey results

Practices	All (*n* = 231, 100%)
Do you cover doors and windows with metallic or cloth webs?, *n* (%)	
Always	46 (19.9)
Frequently	12 (5.2)
Occasionally	12 (5.2)
Rarely	8 (3.5)
Never	153 (66.2)
Do you fumigate in and outside of your home?, *n* (%)	
Always	11 (4.8)
Frequently	20 (8.7)
Occasionally	45 (19.5)
Rarely	48 (20.8)
Never	107 (46.3)

To explore perceptions among women at risk of maternal–infant transmission, we conducted a subgroup analysis of 97 female respondents of childbearing age (mean age: 31 years, SD = 8). Knowledge levels regarding CD were generally favorable: 81% correctly identified vector-borne transmission, and 80% recognized the potential for cardiac complications. However, only 16% were aware of congenital transmission, and just over half (52%) acknowledged the possibility of asymptomatic infection. In terms of attitudes, 80% expressed a strong desire to be screened, 87% showed interest in attending preventive educational talks, and 79% completely agreed that seeking medical attention after a vector bite was appropriate. Despite this, preventive practices were limited: only 21% consistently used mesh or screens on windows and doors, and 49% reported never fumigating their homes (Supplementary Data 2–5).

## Discussion

This study represents the first seroprevalence investigation in this community, revealing an overall *T. cruzi* seroprevalence of 2%. This rate is markedly lower than previous findings in rural communities of Portuguesa state, which reported a general seroprevalence of approximately 20% in 2003 [33]. This decrease may be attributed to reduced resource allocation since the 1990s for the Chagas Disease Control Program in Venezuela created in 1961, represented by a notable reduction in municipalities’ visits and insecticide use, as seen by the reemergence of malaria and dengue fever within national territory [20, 34]. This program, dismantling in 2012, was associated with an increase in seroprevalence among patients who were < 10 years old in endemic communities within Portuguesa state [23], with the exclusion of Virgen de Coromoto as it was labeled as a nonendemic community. In other regions of Venezuela, including indigenous populations, seroprevalence estimates have ranged between 3% and 6% [35–37]. Such differences may be attributable to improved public awareness of the disease and variations in study methodologies, rather than to maternal screening, which has been less systematic in the last decade [38]. It was not possible to make a comparison based on ecological data with the reference study, as it did not specify which part of Portuguesa state was sampled [33].

Our findings on seroprevalence are comparable to regional studies from rural and urban areas in Argentina and Brazil with similar geographic characteristics, which have reported prevalences of < 10% [39–41]. In contrast, other authors describe that indigenous communities from Latin American countries tend to present higher seroprevalence rates, reaching up to 44% in some areas of the Gran Chaco region [42–45], emphasizing ecological difference between studies. It is important to highlight that Bolivian Chagas seroprevalence in the 2010s stabilized around 20% [46].

While our study did not include an active search for infected vectors through entomologic analysis, previous work in the surrounding Virgen de Coromoto communities reported an infection rate of 31% among vectors [47]. However, the advanced age of seropositive individuals (over 60 years) and high antibody avidity suggest that these infections are chronic, indicating a low risk of active transmission within the sampled population.

Regarding knowledge about CD, most participants were familiar with the disease vector, its habitat, and vector-borne transmission (83%). However, only about half recognized blood transfusion as a transmission route, and fewer than one quarter were aware of congenital transmission. These findings align with previous studies [30–32, 48, 49], but contrast with other reports that noted higher awareness of non-vectorial transmission routes [50]. This discrepancy may reflect differences in the effectiveness of public health policies for disease prevention. Additionally, the majority of respondents demonstrated adequate knowledge regarding the cardiac complications associated with CD, similar to observations in Colombia and Panama [30, 31, 49]. Notably, over half of the participants were aware that CD may be asymptomatic—a higher proportion than reported among Latin American migrants in Germany [32].

Attitudes toward CD were generally positive. More than 90% of respondents expressed willingness to undergo voluntary testing, a result consistent with studies from Colombia, Ecuador, and Mexico [30, 31, 50, 51]. Furthermore, 70% indicated that if they encountered a triatomine, they would submit it to a health center for examination. Although this level of acceptance for health center evaluation following a vector bite is similar to findings in Colombia and Mexico [30, 31, 50], it differs from patterns observed in rural Ecuador [51], and may reflect greater confidence in the local health system’s capacity to manage CD.

Despite favorable knowledge and attitudes, preventive practices were suboptimal. Approximately two thirds of respondents did not use screens on doors and windows, and nearly half did not fumigate their homes. This paradox may be influenced by factors such as risk perception, limited economic resources, and inadequate implementation of public health policies [31, 52, 53]. Similar sociodemographic contexts in Bolivia and Mexico have shown that basic knowledge may sometimes engender a false sense of security, leading to neglect of control measures. Moreover, although interventions such as bed nets and window screens are effective, their cost may hinder mass implementation [53, 54]. Additionally, fumigation is traditionally viewed as a state responsibility, and inconsistent governmental practices since the 2000s have contributed to a reliance on state intervention rather than proactive community action [55].

Subgroup analysis of women of childbearing age revealed several important gaps and opportunities for targeted interventions. Awareness of congenital transmission remained notably low (16%), which is concerning given that vertical transmission is now a major route of *T. cruzi* infection in nonendemic and periendemic areas [2]. Similar patterns have been observed in Ecuador, where only 16% of women giving birth were aware of CD, despite many recognizing the vector [56]. Low educational attainment has consistently been associated with higher risk of infection in pregnant women, with studies in Colombia and El Salvador showing significant dose–response relationships between lower education and seropositivity [57, 58]. While our sample included no seropositive women of childbearing age, the prevalence of incomplete secondary education and limited preventive practices suggests a similar risk profile. In contrast, recent data from urban Venezuela reported a seroprevalence of just 0.33% in pregnant women [59], pointing to regional variability in risk and awareness. In response, structured programs such as the maternal screening initiative in Loja, Ecuador, which promotes multidisciplinary collaboration and early detection, provide a model for improving outcomes [60]. These findings underscore the urgent need for context-specific education and screening strategies targeting women of reproductive age in rural Venezuelan communities.

This study has several limitations. The use of non-random sampling introduces potential selection bias, limiting the generalizability of our prevalence estimates to the broader municipality and state. Although the KAP survey was developed by field experts, we were unable to establish definitive cut-off points for categorizing high versus low knowledge, positive versus negative attitudes, or adequate versus inadequate practices, thereby restricting multivariate analyses. Finally, the anonymity of the participants precluded correlation of individual KAP survey responses with seroprevalence data.

## Conclusions

The observed seroprevalence in Virgen de Coromoto community was low compared with rates reported in previous studies. Notably, all seropositive patients were over 60 years of age, suggesting that active disease transmission is currently absent in the area. Although community members demonstrated good knowledge of and positive attitudes toward CD, their preventive practices were inadequate. These findings underscore the need to strengthen efforts by governmental and non-governmental organizations through the development and implementation of integrated public health and vector control policies for the long-term management of CD. Moreover, probabilistic sampling studies across different regions of the state are necessary to further elucidate the epidemiology of CD in Portuguesa state.

## Supplementary Information

Additional File 1

Additional File 2

Additional File 3

Additional File 4

Additional File 5

## Data Availability

All data and materials in this article are included in the manuscript.

## Abbreviations

CD: Chagas disease
KAPs: Knowledge, attitudes, and practices
ELISA: Enzyme-linked immunosorbent assay
SD: Standard deviation
CI: Confidence interval
IQR: Interquartile range
