# Supplementary material for: Prevalence, knowledge, attitudes, and practices regarding Chagas disease in Guanare, Venezuela: a cross-sectional study
Source: Parasit Vectors. 2025 Jun 8;18:215. doi: 10.1186/s13071-025-06846-4 (PMC12147284; doi:10.1186/s13071-025-06846-4)
Supplement: Supplementary file 1 — Additional File 1 [file 13071_2025_6846_MOESM1_ESM.docx]

**Supplementary Data 1.** Laboratory results of patients with *Trypanosoma cruzi* infection

| Sex | Age | Occupation | Community | OD | Avidity (%) |
| --- | --- | --- | --- | --- | --- |
| Male | 62 | Farmer | Virgen de Coromoto | 0.797 | 69 |
| Male | 62 | Construction worker | Virgen de Coromoto | 0.306 | 79 |
| Female | 64 | Housekeeper | Guanare | 1.061 | 83 |
| Male | 66 | Farmer | Virgen de Coromoto | 0.355 | 37 |
| Male | 69 | Construction worker | Virgen de Coromoto | 1.006 | 92 |
| Female | 74 | Housekeeper | Virgen de Coromoto | 1.079 | 88 |
| Male | 75 | Retired | Virgen de Coromoto | 0.938 | 79 |
