# Supplementary material for: Prevalence, knowledge, attitudes, and practices regarding Chagas disease in Guanare, Venezuela: a cross-sectional study
Source: Parasit Vectors. 2025 Jun 8;18:215. doi: 10.1186/s13071-025-06846-4 (PMC12147284; doi:10.1186/s13071-025-06846-4)
Supplement: Supplementary file 2 — Additional File 2 [file 13071_2025_6846_MOESM2_ESM.docx]

**Supplementary Data 2.** Demographic and epidemiological characteristics among women of childbearing age who answered the survey on KAPs

| **Characteristics** | **All (*n* = 97, 100%)** |
| --- | --- |
| Mean age (SD), years | 31 (8) |
| Sex, *n* (%) |  |
| Female | 97 (100) |
| Highest educational degree, *n* (%) |  |
| None | 2 (2.1) |
| Primary school | 20 (20.6) |
| High school | 45 (46.4) |
| University | 30 (30.9) |
| Occupation, *n* (%) |  |
| Housekeeper | 49 (50.5) |
| Construction worker | 6 (6.2) |
| Teacher | 14 (14.4) |
| Administrator | 8 (8.2) |
| Student | 13 (13.4) |
| Businessman | 4 (4.1) |
| Chef | 3 (3.1) |
| Have you seen the kissing bug (chipo) at home?, yes (%) | 35 (36.1) |
| Have you ever been bitten by the bug?, yes (%) | 5 (5.2) |
| After the bite, Did you attend a healthcare facility?, yes (%) | 1 (20) |
| Were you diagnosed with Chagas disease?, yes (%) | 0 (0) |
| Household living surroundings, yes (%) |  |
| Vegetation | 83 (85.6) |
| Pets | 75 (77.3) |
| Common opossum | 65 (67) |
| Palms | 49 (50.5) |
| Have you ever received a transfusion?, yes (%) | 16 (16.5) |
| Have you ever donated blood?, yes (%) | 14 (14.4) |
| Have you ever had a transplant?, yes (%) | 0 (0) |
| Do you frequent natural juices out of home?, yes (%) | 55 (56.7) |
| Do you eat food derived from palms?, yes (%) | 77 (79.4) |
| Do you have family members with diagnosis of Chagas disease?, yes (%) | 27 (27.8) |
