# Supplementary material for: Prevalence, knowledge, attitudes, and practices regarding Chagas disease in Guanare, Venezuela: a cross-sectional study
Source: Parasit Vectors. 2025 Jun 8;18:215. doi: 10.1186/s13071-025-06846-4 (PMC12147284; doi:10.1186/s13071-025-06846-4)
Supplement: Supplementary file 3 — Additional File 3 [file 13071_2025_6846_MOESM3_ESM.docx]

**Supplementary Data 3.** Knowledge survey results among women of childbearing age

| **Knowledge** | **All (*n* = 97, 100%)** |
| --- | --- |
| How is Chagas disease transmitted?, correct (%) |  |
| Through bugs (vectors) | 79 (81.4) |
| From mother to child during delivery | 15 (15.5) |
| Through blood transfusion | 49 (50.5) |
| Through sexual relationships | 15 (15.5) |
| Through physical contact with cats or dogs | 31 (32) |
| Is it possible to acquire Chagas disease without presenting symptoms?, correct (%) | 50 (51.5) |
| Can Chagas disease cause heart enlargement?, correct (%) | 78 (80.4) |
| Is the vector for Chagas disease known as «chipo» in Venezuela?, correct (%) | 84 (86.6) |
| The following represent characteristics for vector refugial at your home: palm roofs, mud walls, crackles in the walls?, correct (%) | 77 (79.4) |
