# Supplementary material for: Prevalence, knowledge, attitudes, and practices regarding Chagas disease in Guanare, Venezuela: a cross-sectional study
Source: Parasit Vectors. 2025 Jun 8;18:215. doi: 10.1186/s13071-025-06846-4 (PMC12147284; doi:10.1186/s13071-025-06846-4)
Supplement: Supplementary file 4 — Additional File 4 [file 13071_2025_6846_MOESM4_ESM.docx]

**Supplementary Data 4.** Attitude survey results among women of childbearing age

| **Attitudes** | **All (*n* = 97, 100%)** |
| --- | --- |
| Chagas disease is severe, *n* (%) |  |
| Completely agree | 53 (54.6) |
| Agree | 21 (21.6) |
| Neutral | 16 (16.5) |
| Disagree | 6 (6.2) |
| Completely disagree | 1 (1) |
| Would you like to be screened and tested for Chagas disease?, *n* (%) |  |
| Completely agree | 78 (80.4) |
| Agree | 19 (19.6) |
| Neutral | 0 (0) |
| Disagree | 0 (0) |
| Completely disagree | 0 (0) |
| If you find the disease vector (Chipo) at home, would you take it to the nearest medical facility?, *n* (%) |  |
| Completely agree | 51 (52.6) |
| Agree | 19 (19.6) |
| Neutral | 10 (10.3) |
| Disagree | 7 (7.2) |
| Completely disagree | 10 (10.3) |
| Attending lectures on the disease could prevent you from acquiring Chagas disease?, *n* (%) |  |
| Completely agree | 61 (62.9) |
| Agree | 23 (23.7) |
| Neutral | 5 (5.2) |
| Disagree | 7 (7.2) |
| Completely disagree | 1 (1) |
| The best next step following a bite from the Chagas disease vector is to attend the nearest medical facility?, *n* (%) |  |
| Completely agree | 77 (79.4) |
| Agree | 13 (13.4) |
| Neutral | 3 (3.1) |
| Disagree | 3 (3.1) |
| Completely disagree | 1 (1) |
