# Supplementary material for: Prevalence, knowledge, attitudes, and practices regarding Chagas disease in Guanare, Venezuela: a cross-sectional study
Source: Parasit Vectors. 2025 Jun 8;18:215. doi: 10.1186/s13071-025-06846-4 (PMC12147284; doi:10.1186/s13071-025-06846-4)
Supplement: Supplementary file 5 — Additional File 5 [file 13071_2025_6846_MOESM5_ESM.docx]

**Supplementary Data 5.** Practice survey results among women of childbearing age

| **Practices** | **All (*n* = 97, 100%)** |
| --- | --- |
| Do you cover doors and windows with metallic or cloth webs?, *n* (%) |  |
| Always | 20 (20.6) |
| Frequently | 5 (5.2) |
| Occasionally | 4 (4.1) |
| Rarely | 1 (1) |
| Never | 67 (69.1) |
| Do you fumigate in and outside of your home?, *n* (%) |  |
| Always | 5 (5.2) |
| Frequently | 8 (8.2) |
| Occasionally | 19 (19.6) |
| Rarely | 18 (18.6) |
| Never | 47 (48.5) |
